# Editorial: Flavor chemistry of food: mechanism, interaction, new advances

**DOI:** 10.3389/fnut.2023.1243606

**Published:** 2023-07-04

**Authors:** Mingquan Huang, Gang Fan, Yanyan Zhang

**Affiliations:** ^1^Key Laboratory of Brewing Molecular Engineering of China Light Industry, Beijing Technology & Business University (BTBU), Beijing, China; ^2^Key Laboratory of Environment Correlative Dietology, Ministry of Education, College of Food Science and Technology, Huazhong Agricultural University, Wuhan, China; ^3^Department of Flavor Chemistry, Institute of Food Science and Biotechnology, University of Hohenheim, Stuttgart, Germany

**Keywords:** flavor chemistry, aroma, mechanism, interaction, volatile compounds, fermentation, storage

Flavor quality is one of the most important qualities of food, including taste and odor. The related investigations on food flavor have been widely reported during the past several decades, which includes multiple aspects, such as new methods and technologies for the analysis of flavor compounds in food; the qualitative and quantitative analysis of the flavor compound in food; the regulation and formation mechanisms of flavor compounds in food during processing and preservation; the interaction between flavor components, macromolecules, and flavor components; the interaction between flavor molecules and olfactory receptors or taste receptors. Due to the diversity and extremely low content of flavor compounds and the complexities of the formation mechanisms of flavor compounds in food, the researches on flavor compounds in food still gain worldwide attention. The goal of this special edition Research Topic was to shed light on mechanisms of metabolism, regulation or formation of key flavor components in food, the interaction between flavor components, food matrix or macromolecules and flavor components, as well as establishing new methods and technologies for the analysis of flavor compounds in food. Additionally, the effects of beer, wine, and baijiu consumption on non-alcoholic fatty liver disease, and the flavor and key influencing factors of Maillard reaction products were reviewed.

Yan et al. established a new ultraperformance liquid chromatography (UPLC) strategy that has been developed for the identification and quantification of volatile thiols in Chinese liquor (Baijiu).

The effect of different varieties on the food flavor has been investigated. Sun et al. studied the volatile constituents and odorous compounds in peach (*Prunus persica* L) fruits of different varieties. Guo F. et al. studied the sensory characteristics and aroma components of nine different fragrant rapeseed oils.

Regarding the volatile compounds in fermented food, Cao et al. investigated the effects of alcoholic fermentation on the non-volatile and volatile compounds of “Cocktail” grapefruit juice. To analyze the latter, a non-targeted metabolomics method based on UPLC-MS/MS and volatiles analysis using GC-IMS were performed. Zhou X. et al. investigated the effects of post-fermentation on the flavor compounds formation in red sour soup. Wang Ya. et al. studied the sensory characteristics of Petit Manseng wine. Its flavor was evaluated by detecting the primary organic acids, phenolic acid compounds, and volatile ester compounds. The authors found that indigenous yeast can increase the phenolic acid and volatile ester compounds in Petit Manseng wine. Pei et al. characterized the transcriptomic profiles and the change of main flavor substance of *Z. rouxii* under salt treatment. Ao et al. set up a method for the quick classification of strong-aroma types of base Baijiu using potentiometric and voltammetric electronic tongue combined with chemometric techniques.

The effect of processing and cooking on food flavor has also been investigated. Wang Yu. et al. studied the effects of four cooking methods on flavor and sensory characteristics of scallop muscle. Wang Y.-R. et al. evaluated the key aroma compounds and protein secondary structure in the roasted Tan mutton during the traditional charcoal process. Zhou M. et al. compared the changes in the quality characteristics of air-fried shrimp meat and deep-fried shrimp meat at different frying temperatures (160, 170, 180, and 190°C). Fei et al. studied the changes in volatile flavor substances during Crataegi fructus roasting using E-nose and HS-GC-MS. Xu et al. investigated the effects of different managements and shade types on the aroma and color generation of roasted coffee beans. Gao et al. investigated the changes of nucleotides, succinic acid, and free amino acids amounts in yolk and the causes leading to the changes after pickling to uncover the fundamental umami component of preserved egg yolk. Guo J. et al. studied the effects of 1% (w/v) alcohol denatured soybean protein isolates, native soybean protein isolates, as well as the thermal denaturation of soybean protein isolates on low concentration (24 μmol/L) of citral in aqueous using headspace solid-phase microextraction/gas chromatography–mass spectrometry.

Storage is an important factor affecting the flavor of food. Zhang et al. studied the effects of three antioxidants, tert-butylhydroquinone (TBHQ), tea polyphenol (TP), and L-ascorbyl palmitate (L-AP), on volatile components, physicochemical properties, and antioxidant activities of heat processed beef flavor over 168 days at different temperatures (4, 20, and 50°C). Zeng et al. studied the effects of methyl salicylate pre-treatment on the volatile profiles and key gene expressions in tomatoes stored at low temperature. Bi et al. studied the effect of high freezing rate on flavor fidelity of hand grab mutton after short-term frozen storage. Wang Z. et al. studied the flavor evolution and important aroma components during long-term storage of compressed white tea using flavor wheel, headspace gas chromatography ion mobility spectroscopy, chemometrics.

In addition, Liu N. et al. found that mild salinity improves the growth, nutrition, and flavor properties of hydroponic Chinese chive (Allium tuberosum Rottler ex Spr). Zhou Y. et al. evaluated and discussed the current human-based and laboratory-based study evidence of effects on hepatic lipid metabolism and non-alcoholic fatty liver disease from ingested ethanol, the polyphenols in beer and wine, and the bioactive flavor compounds in baijiu, and their potential mechanism. Liu S. et al. mainly reviewed the Maillard reaction-derived flavors, the main substances producing Maillard reaction-derived flavors, and the detection methods were also introduced.

In conclusion, we hope this article Research Topic can provide new information in the field of flavor chemistry of food.

## Author contributions

GF wrote the initial draft of the manuscript. MH and YZ finalized the manuscript. All authors contributed to the article and approved the submitted version.

